# Acute lung toxicity from nitrofurantoin in an immunosuppressed patient

**DOI:** 10.1097/EA9.0000000000000042

**Published:** 2023-12-06

**Authors:** Maria Madalena Nabais, Lionel Müller, Sylvain Picard, Sebastian Schramm

**Affiliations:** From the Department of Anesthesiology (MMN, SS), Department of intensive Care, Hôpital Cantonal de Fribourg, Fribourg (LM) and Department of Intensive Care, Spitalzentrum Biel, Biel, Switzerland (SP)

In April 2021, an immunosuppressed woman in her 50s presented to the emergency department with an acute onset of shortness of breath. She has given consent for this publication. Her past medical history revealed a kidney and pancreas transplant for type I diabetes and chronic renal failure in 2019. She was taking mycophenolate mofetil and tacrolimus as immunosuppressive therapy. Due to a lower urinary tract infection, 3 days previously she had been commenced on oral nitrofurantoin. She was conscious, febrile (38.6 °c), with blood pressure 120/74 mmHg, tachycardic 108 beats minute^−1^, tachypnoeic 24 breaths minute^−1^ and oxygen saturation 74% in room air. Auscultation of the lungs revealed diffuse fine crackles with basilar hypoventilation. Arterial blood gases with high-flow oxygen mask (FiO_2,_ 42%) were pH 7.39, *P*aO_2_ 14.6 kPa, *P*aCO_2_ 4.5 kPa and HCO_3_ 20 mmol l^−1^. The *P*aO_2_ /FiO_2_ was 261 mmHg. Her peripheral white cell count was 9.2 g l^−1^ with 7.2 g l^−1^ polynuclear neutrophils. Eosinophilia was absent. C-reactive protein was 104 mg dl^−1^ with creatinine stable at 141 **μ**mol l^−1^ and hepatic function was normal. The electrocardiogram was normal. The echocardiogram showed a left ventricular ejection fraction 57% (normal, *Simpson method*), with signs of moderate left ventricular hypertrophy and diastolic dysfunction grade I, suggesting hypertensive heart disease in a patient with known long-standing renal disease. The mitral *E* wave velocity was 55 cms^−1^, *E*/*A* at ratio of 0.67 and *E*/Ea at ratio of 6.49. NT-proBNP was 498 ng ml^−1^. Chest radiography showed bilateral alveolar infiltrates (Fig. 1). A CT lung scan and pulmonary ventilation/perfusion scan showed widespread bilateral ground-glass alveolar infiltrates without evidence of pulmonary embolism. Intravenous ertapenem was started because of the urinary infections with *Klebsiella pneumoniae ESBL*. Unfortunately, her respiratory status declined over 24 h and she was transferred to the intensive care unit (ICU) where she was intubated for severe hypoxemia and, despite adequate volume resuscitation, she required vasopressors. Initial ventilatory settings in the ICU included FiO_2_ between 30 and 45%, low positive end-expiratory pressure at 4 cmH_2_O, and tidal volumes between 370 and 450 ml.

**FIGURE 1:**
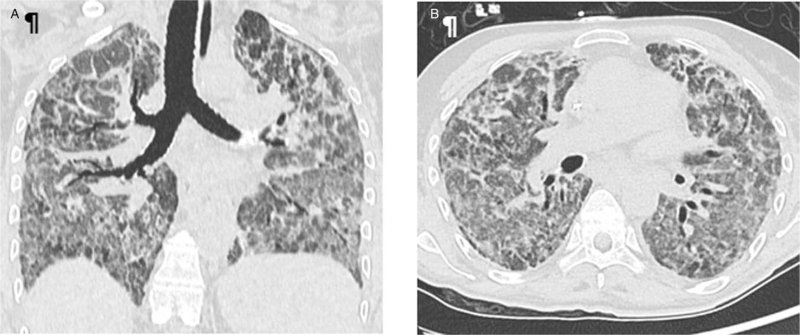
(a and b) CT lung scan: bilateral widespread ground-glass patches associated with interlobar thickenings, and banded consolidation. Note areas of parenchymal sparing, particularly at the apex.

## Investigations

Two pairs of blood cultures were negative for bacterial infection. Urine culture and faecal samples were positive for *K. pneumoniae ESBL*. Urinary antigens for *Legionella pneumophila* and *Streptococcus pneumoniae* were negative. Faecal sample testing was negative for *Clostridium difficile*. Bronchoalveolar lavage analysis was performed and PCR-viral testing was negative for *SARS-Cov-2,* Respiratory syncytial virus, *Pneumocystis carinii,* and *Influenza A* and *B.* Fungal and tuberculosis tests were negative. Cell counting and cytopathology analysis of bronchoalveolar lavage showed normal cellularity (180 elements per microlitre), absence of malignancy features and neutrophilic alveolitis (60% neutrophil granulocytes, 31% macrophages, 1% lymphocytes) with a gold score of 80.

## Differential diagnosis

Nitrofurantoin pulmonary toxicity is mainly a diagnosis of exclusion. Healthcare-associated pneumonia, viral/fungal infections in immunocompromised hosts, and intra-alveolar haemorrhage were excluded by bronchoalveolar lavage. Pulmonary embolism was excluded by the ventilation-perfusion scan. Graft-versus-host acute reaction was excluded by the onset timeline of more than 100 days after transplantation. The chronic form of this disease has an insidious onset and is typically diagnosed in bone marrow transplant patients presenting with a skin rash and liver injury.^1^ Lung involvement in systemic vasculitis was not investigated because of absence of multiorgan injury.^2^ Arguments supporting acute nitrofurantoin-induced pulmonary toxicity are past medical drug exposure, common acute clinical manifestations such as fever, cough and dyspnoea, radiological changes and fast recovery after drug withdrawal. In addition, the Naranjo adverse drug reaction probability scale scored four points indicating a possible reaction.^3^ Against our diagnosis is the absence of eosinophilia in laboratory tests, which is often a common finding in these patients.^4^ Lung biopsy would have been useful to exclude cryptogenic organising pneumonia and interstitial lung disease. Lastly, cardiogenic pulmonary oedema cannot be fully excluded: slightly elevated NT-proBNP, mild diastolic dysfunction and widespread ground-glass bilateral infiltrates are described in CT lung scan.

## Treatment

Supportive treatment was immediately instituted. Oral nitrofurantoin was stopped, and a 1-week course of the antibiotic ertapenem intravenously was commenced. A tapering steroid regimen over 6 weeks was also started. After discussion with the transplantation team, mycophenolate mofetil treatment was suspended briefly and the tacrolimus dose was increased as target blood levels were low.

## Outcomes and follow-up

Her clinical condition improved and the vasopressors were stopped after 4 days, with tracheal extubation after 7 days. She was transferred to a medical ward and was completely weaned off oxygen at hospital discharge. In the hospital outpatient department 14 months later, her SpO_2_ in room air was 98%.

## Discussion

Nitrofurantoin is a widely prescribed antibiotic for treating lower urinary tract infections, as well as prophylactic treatment against recurrent lower urinary tract infections. It is taken up by bacteria, where intracellular flavoproteins reduce it to reactive intermediates, which bind to bacterial ribosomes, inhibiting various bacterial enzymes, especially those involved in synthesis of DNA, RNA and cell wall proteins.^5^ It is absorbed from the gastrointestinal tract, metabolised by the glutathione S-reductase system in the liver and is mainly excreted in the urine.^6^ The mechanism of pulmonary-induced toxicity remains unknown.^7^

Nitrofurantoin pulmonary drug side-effects are rare but can result in life-threating conditions with prolonged hospitalisation. Pulmonary disorders associated with nitrofurantoin toxicity include cryptogenic organising pneumonia, diffuse alveolar haemorrhage, and acute, sub-acute or chronic interstitial lung disease.^6^ The gold standard treatment remains withdrawal of the drug and supportive treatment. Steroids are often prescribed but their benefits remain unclear.^6^

Concerning patient safety, physicians should identify patients at risk of nitrofurantoin toxicity before starting treatment. Patients should be informed of the potential toxicities, and clinically monitored for signs and symptoms at regular intervals.^7^ Identifying past drug exposure in respiratory symptomatic patients is also key in avoiding misdiagnoses. Reporting these cases to pharmaco-vigilance health institutions increases available data on patient morbidity and mortality, contributing to better healthcare decisions in the future.
